# Ocean Planning and Conservation in the Age of Climate Change: A Roundtable Discussion

**DOI:** 10.1093/iob/obae037

**Published:** 2024-09-26

**Authors:** C Frazão Santos, T Agardy, L B Crowder, J C Day, A Himes-Cornell, M L Pinsky, J M Reimer, E Gissi

**Affiliations:** Department of Animal Biology, Faculdade de Ciências, Universidade de Lisboa, 1749-016 Lisbon, Portugal; MARE–Marine and Environmental Sciences Center/ARNET–Aquatic Research Network, Universidade de Lisboa, 1749-016 Lisbon, Portugal; School of Geography and the Environment, University of Oxford, Oxford OX1 3QY, UK; Sound Seas, Bethesda, MD 20816, USA; Oceans Department, Hopkins Marine Station, Stanford University, Pacific Grove, CA 93959, USA; College of Science & Engineering, James Cook University, Townsville, QLD 4815, Australia; Fisheries and Aquaculture Division, Food and Aquaculture Organization of the United Nations, 00153 Rome, Italy; Department of Ecology & Evolutionary Biology, University of California Santa Cruz, Santa Cruz, CA 95060, USA; Department of Ecology, Evolution and Natural Resources, Rutgers University, New Brunswick, NJ 08901, USA; Department of Geography, Memorial University of Newfoundland and Labrador, St. John's, NL A1C 5S7, Canada; Marine Planning & Conservation, Fisheries and Oceans Canada, Ottawa, ON K1A 0E6, Canada; Oceans Department, Hopkins Marine Station, Stanford University, Pacific Grove, CA 93959, USA; National Research Council, Institute of Marine Sciences, 30122 Venice, Italy; National Biodiversity Future Center, 90133 Palermo, Italy

## Abstract

Over recent years, recognition of the need to develop climate-smart marine spatial planning (MSP) has gained momentum globally. In this roundtable discussion, we use a question-and-answer format to leverage diverse perspectives and voices involved in the study of sustainable MSP and marine conservation under global environmental and social change. We intend this dialogue to serve as a stepping stone toward developing ocean planning initiatives that are sustainable, equitable, and climate-resilient around the globe.

## Synopsis (Portuguese)

A necessidade de desenvolver processos de ordenamento do espaço marinho (OEM) que sejam inteligentes do ponto de vista climático tem sido amplamente reconhecida internacionalmente nos últimos anos. Na presente mesa-redonda, adoptou-se um formato de pergunta-resposta para promover uma discussão sobre os desafios envolvidos na conservação dos ecossistemas marinhos e no desenvolvimento de planos de OEM sustentáveis, num contexto de alterações ambientais e sociais globais. Foram debatidas diversas perspectivas e ouvidas várias vozes, com o objetivo de contribuir para um OEM sustentável, equitativo e resiliente às alterações climáticas a nível global.

## Synopsis (Spanish)

En los últimos años se ha reconocido internacionalmente la necesidad de desarrollar procesos de planificación espacial marina (PEM) climáticamente inteligentes. En esta mesa redonda se utiliza un formato de preguntas y respuestas para promover una discusión sobre los desafíos que implica la conservación de los ecosistemas marinos y el desarrollo de PEM sostenibles en un contexto de cambios ambientales y sociales globales. Se presentaron y debatieron diversas perspectivas y voces con el objetivo de contribuir a una PEM sostenible equitativa y resiliente al clima a nivel global.

## Introduction

Over the past decades, marine spatial planning (MSP) has spread widely as a way to balance sustainable ocean use and conservation (Ehler 2021). Formal MSP initiatives are under development in over 120 countries/territories around the globe (United Nations Educational, Scientific, and Cultural Organization [UNESCO] 2024) and will keep expanding across latitudes and ocean basins, and from national waters to the high seas (Frazão Santos et al. 2024a). Being a process of analyzing and allocating human uses of the ocean in space and time to achieve social, economic, and ecological objectives, the effectiveness of MSP development and implementation has faced multiple challenges—from institutional settings to knowledge availability to stakeholder engagement (Frazão Santos et al. 2021). On top of these challenges, and with high potential to significantly exacerbate them, is climate change (IPCC 2019, 2023).

Climate-induced changes in marine ecosystems and oceanographic features will affect the ways people relate to and benefit from the ocean. Areas where human activities are most amenable to take place today will likely be different in the near future. To respond to these changes while effectively supporting sustainable and equitable ocean use, MSP initiatives will need to be “climate-smart” (i.e., integrate climate-related knowledge, be flexible and adapt to changing conditions, and support climate adaptation and mitigation actions) (Frazão Santos et al. 2020, 2024b).

Until now, few existing marine spatial plans have explicitly considered climate change, which is a critical oversight in a rapidly changing world. However, over recent years, recognition of the need to effectively develop climate-smart MSP has gained momentum globally. The UNESCO and the European Commission launched a joint MSP roadmap in late 2022, acknowledging the development of climate-smart MSP as one of the six key priority areas for 2022–2027 (UNESCO and European Commission 2022). Other institutions like the World Bank, the United Nations Global Compact, or the International Council for the Exploration of the Sea have also produced international documents (United Nations Global Compact 2021; World Bank 2021a; ICES 2024) and developed initiatives to support the topic's discussion (Table 1).

**Table 1.** Key events and publications by international institutions on the nexus between marine spatial planning and climate change

Institution(s)	Event	Link
OCTO-Open Communications for the Ocean	*Webinar*, “The effects of climate change in marine spatial planning: Pathways and solutions,” October 2020	https://octogroup.org/the-effects-of-climate-change-in-marine-spatial-planning-pathways-and-solutions/
MARE-Center for Maritime Research	*Conference Session*, “Session 3.161: Marine Spatial Planning and Ocean Conservation in the Age of Climate Change,” July 2021, 11th MARE People and the Sea Conference	https://marecentre.nl/2021-conference/
The World Bank	*Webinar*, “Marine Spatial Planning Webinar Series: Climate Informed Planning and Implementation,” June 2021	Not available
VASAB and SwAM	*Conference Session*, “Workshop 9: MSP and Climate Change,” June 2021, 4th Baltic Marine Spatial Forum	https://vasab.org/4th-baltic-msp-forum/
ICES	*Conference Session*, “Theme Session B: Spatial Management, Climate Change and Biodiversity,” September 2022, ICES Annual Science Conference	www.ices.dk/events/asc/ASC2022/Pages/Theme-session-B.aspx
UNESCO and European Commission	*Conference Session*, “Session 6: Climate-smart MSP,” November 2022, 3rd UNESCO/European Commission International Conference on Marine/Maritime Spatial Planning	www.mspglobal2030.org/msp-roadmap/msp-conference/3rd-intl-conf-msp/
ICES, PICES, UNESCO, and FAO	*Conference Session*, “Session 1: Marine Spatial Management Supporting Climate Change Adaptation and Mitigation,” April 2023, 5th International Symposium on Effects of Climate Change on the World's Oceans	https://meetings.pices.int/meetings/international/2023/eccwo-5/program#S1
ICES	*Workshop*, “Workshop on Climate Change Considerations in Marine Spatial Planning,” October 2023	www.ices.dk/community/groups/Pages/WKCCCMSP.aspx
OCTO-Open Communications for the Ocean	*Webinar*, “Taking Climate-Smart Ocean Planning and Governance to the High Seas,” July 2024	https://octogroup.org/taking-climate-smart-ocean-planning-and-governance-to-the-high-seas/
ICES	*Conference Session*, “Theme Session I: Accounting for Climate Change in Marine Spatial Planning: Experiences and lessons Learnt,” September 2024, ICES Annual Science Conference	www.ices.dk/events/asc/2024/Pages/Theme-session-I.aspx
UNESCO and European Commission	*Conference Session*, “Session 3: MSP & Climate Change,” October 2024, 6th International MSP Forum	www.mspglobal2030.org/msp-forum/bali/
UNESCO	MSPglobal Policy Brief: Climate Change and Marine Spatial Planning. *Intergovernmental Oceanographic Commission Policy Brief no. 3* (2021)	https://unesdoc.unesco.org/ark:/48223/pf0000375721
The World Bank	Climate-Informed Marine Spatial Planning. *PROBLUE Knowledge Factsheet Series no. 2* (2021).	www.worldbank.org/en/programs/problue/publication/marine-spatial-planning-for-a-resilient-and-inclusive-blue-economy-toolkit
United Nations Global Compact	Roadmap to Integrate Clean Offshore Renewable Energy into Climate-Smart Marine Spatial Planning. *Ocean Stewardship Coalition* (2021).	https://unglobalcompact.org/library/5977
UNESCO and European Commission	Updated Joint Roadmap to accelerate Marine/Maritime Spatial Planning processes worldwide: MSProadmap (2022–2027). *Intergovernmental Oceanographic Commission Technical Series no. 182* (2022).	https://unesdoc.unesco.org/ark:/48223/pf0000385718
ICES	Workshop on Climate Change Considerations in Marine Spatial Planning (WKCCCMSP; outputs from 2023 meeting). ICES Scientific Report no. 6:57 (2024).	https://doi.org/10.17895/ices.pub.25933072

Abbreviations: FAO, Food and Agriculture Organization of the United Nations; ICES, International Council for the Exploration of the Sea; PICES, North Pacific Marine Science Organization; SwAM, Swedish Agency for Marine and Water Management; UNESCO, United Nations Educational, Scientific and Cultural Organization; VASAB, Vision and Strategies Around the Baltic Sea.

Most recently, 10 key components of climate-smart MSP were identified to support marine managers and planners at the practical level, providing guidance on how to put the concept into action (Frazão Santos et al. 2024b), and the potential benefit of implementing such an approach in areas beyond national jurisdiction was highlighted (Frazão Santos et al. 2024a). While the identification of the key components proposed by Frazão Santos et al. (2024b) resulted from multiple conversations and debates, their foundation drew heavily from discussions held during a scientific session at the 11th biennial MARE People & the Sea Conference in 2021—session 3.161, “Marine Spatial Planning and Ocean Conservation in the Age of Climate Change” (Table 1). As the conference was devoted to “Limits to Blue Growth” (MARE 2021), the scientific session promoted a critical debate with scientists and practitioners (details in Supplementary Table 1) around the multiple pathways, challenges, and benefits of developing sustainable MSP initiatives under changing environmental and social conditions, as well as the need for a paradigm shift toward MSP initiatives that truly support the health of coupled human–ocean systems (a healthy ocean being one that sustainably delivers a range of benefits to people now and in the future; Halpern et al. 2012; Nash et al. 2022). Indeed, finding the right balance between human development and environmental protection has been one of the most striking and widespread challenges in MSP development (Trouillet and Jay 2021), and solutions are not straightforward.

Here, we dive deep into the discussions held at the People & the Sea conference scientific session, further unpacking and debating fundamental aspects that were at the origin of the 10 key components of climate-smart MSP (Frazão Santos et al. 2024b). We use an informal question–answer format to discuss these critical topics, and raise awareness close to the scientific community, marine managers and planners, decision-makers, and other stakeholders. To that purpose, original session transcripts were edited to improve text clarity and readability while reflecting the roundtable discussions. Five key topics were addressed: (1) main challenges posed by climate change to MSP; (2) integration of climate-related knowledge into MSP; (3) development of dynamic and adaptive marine spatial plans; (4) ocean health as the foundation for MSP in a changing ocean; and (5) building common narratives to engage stakeholders and decision-makers. We intend this dialogue to promote further insights into a timely topic that plays a key role in the future of our ocean.

## Climate-related challenges and solutions

Many challenges surround the development of MSP under a changing climate. Here, we dive into existing issues and explore potential solutions. Three key topics were addressed to guide this first part of the session: (1) identification of major challenges; (2) integration of climate knowledge; and (3) development of flexible plans.

### What are the biggest challenges that climate change can pose to MSP?

*A key challenge of developing MSP under climate change is managing the inherent uncertainty. We discuss uncertainty, touching on issues ranging from developing predictive models to integrating uncertainty into legal documents. The challenge of ensuring social equity and justice is also raised*.

**T.A.:** The biggest challenge posed by climate change to MSP is the uncertainty it implies. While we clearly need to make MSP more dynamic, responsive, and forward-looking, uncertainty is a major challenge to these goals. We need to think about how to respond to what we know will happen with climate change while considering that we do not have the entire picture, particularly in the context of cumulative effects from other stressors (Gissi et al. 2021). We are starting to understand better how climate change affects overall ecosystem health and productivity (e.g., distributional shifts, changes in productivity, and sex-related impacts on species) (Pecl et al. 2017; Gissi et al. 2023). We now need to build marine spatial plans that are able to deal with the uncertainty that remains and generate the information needed to deal with such uncertainty, building more robust models and better anticipating changes. We must commit to this.

**E.G.:** Indeed, conceiving a vision for the future is a significant challenge for MSP under climate change. As we do not know precisely how the future will unravel, we need to use the knowledge we produce to explore potential “futures” (which are plural). We must use such knowledge to develop dynamic and proactive planning and management, and cope with such an uncertain future. We have the needed tools and are building the knowledge to develop scenarios and imagine the future depending on different climate projections and underlying decision pathways (Swart et al. 2004). Then, in addition to the challenge of building different scenarios for potential futures, we need to navigate them and define robust management strategies to move toward the future we want.

Another major challenge is moving beyond mapping spatial overlaps—now and in the future—and look at the cause–effect relationships behind these dynamics from a functional perspective. We are, in fact, still struggling at more fundamental levels (e.g., in habitat mapping). However, we need to think forward and consider the functionality behind spatial data and maps, finding ways to represent the relationships between multiple causes and related multiple effects (Gissi et al. 2021).

**L.B.C.:** The points raised by Tundi and Elena are very appropriate. A famous quote attributed to Nobel prize-winning physicist Niels Bohr and baseball player Yogi Berra says, “It is difficult to make predictions, especially about the future” (Dickstein 2021). The latter is profound and underlines the difficulties we face when trying to develop MSP that is resilient to expected future changes—especially as we are planning for the future based on data about the past; that is, we are looking forward based on data that is looking backward.

For example, to phase down the use of fossil fuels and mitigate and adapt to climate change, we need to further allocate space to renewable energy production (United Nations Global Compact 2021). As such, MSP will sometimes entail establishing “semi-permanent” installations—nothing is permanent in the ocean—such as offshore wind and tidal energy developments. Wind farms might be placed in what is currently nearshore (for cabling) and where wind fields are strong and most consistent today. However, where will those wind fields be in 50 years? How will the environment around these facilities change over the engineering lifetime of these facilities?

Also, if we have a whale-watching business, but whales are not expected to be anywhere near us in 30 years, we have a problem. To establish a business, we thus need to make predictions about the future. This is true for all resources we depend upon, from tourism to fisheries. With prospective planning, we are trying to make this break between mapping the past and looking a short distance into the future to looking a long distance into the future. While making such predictions is hard, and we have every reason to believe we can be wrong, I am hopeful about some of the new tools and approaches being developed, which allow us to envision how the ocean will likely be in 50 years (e.g., where wind fields and whales are likely to be). The latter should be part of the planning process.

However, developing “traditional” marine spatial plans is already complex (Frazão Santos et al. 2021), and the people who have done it so far are not to be faulted. We are identifying problems that must be addressed to make MSP more robust. But we are also highlighting the tools to solve them, and we are learning from developing these tools and providing key insights about the future. So, we should be optimistic about doing this.

**M.L.P.:** Consistent with what others have said, a challenging aspect is that climate impacts are often associated with high uncertainty. If something is uncertain, it becomes easy to ignore; it is how the human brain works. Still, like Larry, I believe predictions are valuable and relevant to stretching our perceptions of what the future may look like. It is not about a precise prediction—usually unrealistic—but about unraveling the range of possible futures that can take place (Tolvanen et al. 2019; Kelly et al. 2022). We can then use such a range of futures to stress-test our plans or scenario planning. Ultimately, we can assess which marine spatial plans will be robust to the range of futures we are facing.

There is a real contrast between the human need for predictability and static lines on a map, including long-term ocean infrastructures that do not move, and the dynamic nature of biological and oceanographic features. How do we then design for change? How do we make adaptable systems, or systems that are robust to a range of futures, when they cannot adapt through time? This is the most challenging part. We need to use a stress-testing or scenario-planning process to identify plans that will work despite change.

There is also the challenge of social equity (Crosman et al. 2022). Social equity will be further exacerbated by climate change, as climate-related impacts tend to affect marginalized human communities disproportionally compared to those already powerful. In such a context, how do we ensure that the benefits of ocean development and the use of ocean resources are spread widely and not just concentrated among those who hold power in our current society? This is a major challenge (Österblom et al. 2020; von Thenen et al. 2021). Also, the latter partially relates to the fact that social data is very qualitative and, therefore, difficult to integrate into technocratic, data-driven processes such as MSP (Le Cornu et al. 2014; Gilek et al. 2021).

**J.C.D.:** Many people think the Great Barrier Reef in Australia is an exceptional example of MSP (Day et al. 2019). When we talk about large-scale MSP, there are many aspects about which the managers are justifiably proud. However, over the last few decades, the reef has faced marine heatwaves, tropical cyclones (many category 5, the most severe), droughts, and floods. We also had bleaching events in 2016, 2017, and 2020 with a tremendous impact on corals across the reef—in 2016 and 2017 alone, over 50% of the corals suffered severe bleaching, leading to high levels of mortality (GBRMPA 2019; Hughes et al. 2021). There are many different MSP zones across the Great Barrier Reef, and bleaching occurred regardless of the type of zone. Also, climate-related risks in the reef are all rated in the likelihood category of “almost certain,” with moderate to catastrophic consequences—for example, ocean warming and acidification (*catastrophic*), altered weather patterns, sea level rise (*major*), and altered ocean currents (*moderate*) (GBRMPA 2019). This is to say that highly protected areas and no-fishing areas are not immune to climate change. However, we are finding—and time will tell—that these areas are more resilient and better able to recover than partially protected areas (Jacquemont et al. 2022).

As for uncertainty, a fundamental problem from a practical management perspective is that it is extremely difficult to integrate it into a legal document that is to be approved by decision-makers. Decision-makers do not like plans that feature too much flexibility; they prefer something clear and consolidated that they can control (Craig et al. 2017). This often conflicts with the uncertainty inherent to climate change and the dynamic nature of the ocean. It is a major dilemma when we have legal complexity combined with something that will most likely change.

Another important aspect is that many planning initiatives have tried to get everything into a single two-dimensional plan, which is signed off as it goes up the hierarchy. Having a single plan is a less effective way to proceed, as it removes flexibility—and we need flexibility. MSP is not a single plan but a range of layers of integrated plans, and managers need the flexibility to adapt and “move things around” if required. However, the foundation upon which we are planning needs to be “set in stone” so it is not easily changed. Otherwise, if there is a governmental change or a change in ministries, decision-makers can go back and overrule established decisions to fit their ways of thinking (Levin et al. 2019; Wölfl et al. 2019; Frazão Santos et al. 2021). We must ensure enough certainty to allow for effective conservation, the basis upon which we must be doing MSP.

So, integrating uncertainty about what will happen in the future into a legally binding document while ensuring flexibility to allow for change is challenging.

### How can we effectively integrate climate-related knowledge and considerations into MSP?

*The relevance of scenario planning, species distribution modelling, and assessment of cumulative impacts as tools for the proper integration of climate knowledge into ocean planning is discussed here. The potential of blue carbon ecosystems as a climate mitigation approach is also identified*.

**M.L.P.:** Scenario planning is a valuable technique that has become increasingly common in coastal development planning and fisheries management—and could be used more often in MSP. In scenario planning, we lay out a discrete set of disparate but plausible futures (McGowan et al. 2019; Tolvanen et al. 2019). Those can include climate-related impacts as well as human development, social impacts, and social considerations, among others. We found that proactive, future-looking MSP is consistently better at meeting planning goals over time. At the same time, future-looking marine spatial plans do not need significantly more area than those considering current conditions only (e.g., for North America, proactive plans needed only about 2% more) (Pinsky et al. 2020). Moreover, proactive plans are robust to climate-related uncertainty, as well as to El Niño and interdecadal natural variability.

We are starting to get the tools for adapting and integrating these shifts in ocean resources and conditions into MSP, at least for North America. For example, the NOAA Fisheries and the Fisheries and Oceans Canada jointly supported the development of the OceanAdapt website (https://oceanadapt.rutgers.edu) that hosts projections of species distributions in the future, as well as the associated uncertainty range, which is fundamental to consider as well.

We see clearly that marine species are already on the move, and ignoring this will lead to marine spatial plans that function poorly. At the same time, we have the tools and techniques for adapting to these changes. Now is a critical time to think about changing ocean conditions because there is also a massive need for greater renewable energy development to support climate change mitigation (Haggett et al. 2020). Such development will lock us into a new seascape for decades if not centuries. So, thinking about MSP and the future is more important than ever.

**L.B.C.:** About 10 years ago, we engaged in a study where we modeled the distribution of species to predict where animals were likely to be seasonally, as well as in 10, 30, 50, and 100 years from now (Hazen et al. 2013). We used ocean climate models to predict the habitats for 2050 or 2100 and then asked the models where the habitat footprints would likely be for these species. For some oceanic species, habitat declined, while for some tropical species, habitat increased. The key point is that we need to know where habitats and species will likely be in the future to have resilient marine spatial plans. We need to follow those moving oceanographic features and organisms that we are interested in protecting or promoting the use of. The take-home message is that MSP needs to pay attention to the fact that we are trying to protect and use resources that are “on the move.”

**M.L.P.:** Indeed, MSP is based on where species are found. However, marine species are moving rapidly as a result (in part) of climate change and variability. Past species distributions are, therefore, not a good guide for the future. For example, on the northeast coast of the United States, black sea bass expanded 500 km north over four decades (Lenoir et al. 2020). On average, marine species are shifting nearly 60 km per decade, which is five times faster than on land—certainly much faster than we perceive in our daily lives. We expect these shifts to continue or even accelerate over the rest of this century (Morley et al. 2018), and in some cases, species are expected to move up to 1000 km or more. The problem is that, so far, MSP efforts are not set up with this likely future in mind.

Shifts in species distribution have important implications for MSP. We have been testing the design of marine spatial plans and found that those focusing only on where species are now are less likely to meet planning goals and less effective as species move. For example, a consistent decline in meeting MSP goals was observed in nine regions around the United States (Pinsky et al. 2020). This is especially relevant for “non-static” ocean uses and activities, such as marine conservation or fisheries. It is also relevant to avoid conflicts between the latter and more “static” uses, such as renewable energy production, that will find it challenging to adapt dynamically over time (e.g., wind turbines are effectively stuck in place for decades).

However, there are approaches available to address this challenge. One potential solution is to include projections of where species will likely be in the future in plans and select areas for conservation, fisheries, and other ocean uses based on such information. This will be consistently useful through time, offering long-term benefits, and will avoid interactions between incompatible ocean activities. In some cases, we can even do this without dynamic areas, only static ones. When we set up planning areas as stepping stones in the ocean, we find that conservation, fisheries, and other goals continue to be met despite shifts in species distribution (Pinsky et al. 2020).

An additional aspect to consider is that when we design for change, we cannot only think of changing ecological patterns (i.e., species distributions and community composition). We must also consider changing social dynamics. In many cases, social changes are even more dramatic than ecological ones.

**E.G.:** In addition to what Larry and Malin have said, a key challenge for MSP is understanding and integrating the diverse responses of marine organisms and ecosystems to multiple pressures, including climate change, at multiple scales and levels of biological organization. For what concerns ecosystem changes, our knowledge about climate-induced responses of marine organisms suffers from severe taxonomic and geographic biases (Feeley et al. 2017), while species vulnerability to climate change varies as a consequence of life history traits (Butt et al. 2022). Moreover, we also struggle to understand how communities will respond to climate change, given that they will reorganize due to individual and population responses to change (Rilov et al. 2019).

For instance, sex-based intraspecific differences in the response of males and females to warming can influence reproductive capacity and population viability (Gissi et al. 2023). These population effects can cascade at the community level, influencing ecosystem functioning and the benefits we derive from ecosystems. Regarding the combined effects of local human stressors with climate change stressors, research has explored the combined effects of fisheries and temperature increase (Gissi et al. 2021). However, many other combined effects must be explored so that MSP can control induced changes by acting on local anthropogenic stressors. As scientists, we must also focus on understanding the nuanced characteristics of potential climate-induced changes to be able to act on the drivers of change.

**J.C.D.:** To follow up on Elena's comment, we do need to account for cumulative pressures. For almost three decades (1985–2013), about half of the 29 World Heritage-listed coral reef properties around the world were exposed to bleaching at least twice per decade (Heron et al. 2017). Current predictions are that by the end of the century, all World Heritage coral reefs will experience severe annual bleaching and cease to host functioning coral reef ecosystems. If these are “the best of the best” managed and planned coral reefs, what does this mean for the rest of the coral reefs worldwide? Issues such as climate change, water pollution, and coastal development are not just facing coral reefs. The same pressures affect most areas where we are doing MSP. It is a problem of cumulative impacts. While climate change is important, we must work on many fronts to address cumulative impacts (Stelzenmüller et al. 2020; Gissi et al. 2021). In the Great Barrier Reef in Australia, a significant amount of work is being undertaken to build resilience. However, the values within the system are still suffering due to the cumulative impacts of a range of factors, including climate change. This means that even the arguably best-managed marine protected area in the world, with enormous efforts for MSP, is still dealing with this problem. What we are discussing here today is, thus, critical for the future of all marine areas.

**T.A.:** Just one additional point that builds on what Malin has said earlier: We also need to think about the mitigation component. Part of that is blue carbon, which is stored by marine and coastal ecosystems (Bertram et al. 2021; Hilmi et al. 2021). We must convince decision-makers that investing in protecting and restoring potential blue carbon ecosystems makes good business sense. This is one of many tools we need to employ very quickly to mitigate the worst impacts of climate change. So, there is much potential there.

**M.L.P.:** Blue carbon sequestration is a very important ocean use going forward, as Tundi mentioned. However, we are still in the process of understanding how durable and long-lasting different sequestration options are and how much carbon they sequester. So, this needs to be connected to monitoring and ongoing research (Macreadie et al. 2017, 2019). This implies that feedback from the process of “doing it” is essential.

### Do we need to develop MSP that is adaptable and flexible to change?

*Yes. Here, we discuss several challenges of developing adaptive and flexible marine spatial plans, which can properly respond to complex systems’ dynamics. We further identify adaptive management and governance, dynamic ocean management, and monitoring and evaluation as key management approaches moving forward*.

**T.A.:** Adaptive management is key. We need to think about MSP as less of a product and more of a process. We must move away from the idea that MSP is about producing a plan; it is not. It is about producing a process, a living process that goes on forever (Douvere and Ehler 2011) and will continue to return benefits to those who invest in it over time.

Also, good MSP, effective MSP, is not only “marine” spatial planning. It is marine, coastal, and watershed, and terrestrial spatial planning. It is about incorporating land use, watershed, coastal zone management, and thinking about the existing interconnections. Furthermore, it is about considering climate change and how it will affect all the elements interlinked with ocean health. For example, what must we do in estuaries affected by climate change to boost the management and conservation of key species, habitats, and ecosystem processes? What must we do to influence land use to maintain ocean health? We must think through these connections. Thinking about how a marine spatial plan can influence the protection of linked habitats or even guide the restoration of degraded habitats to achieve optimal resilience (Manea et al. 2023) is fundamental to going forward in a climate-modified future.

We, as scientists, need to bring to the table the notion that we must be flexible, adaptive, and integrative; we need to think about connections, incorporate social sciences with ecological sciences, and do everything in a truly effective way. It is very complex. However, humans have learned to deal with complex systems. For example, we can think about how there is a lot of complexity and uncertainty in endeavors such as those in the business community and how humans manage to deal with such uncertainties and complexities and to adapt. There is great potential in harnessing MSP to its maximum ability to anticipate changes that are coming, lessen our impact on ecosystems, and ensure that benefits continue to flow as widely as possible. While it is complicated, it is doable.

**E.G.:** When thinking about adaptive strategies, we should consider being “incremental.” We should look at processes and try to build windows of opportunity, leveraging where we have an entry point. For example, we could integrate knowledge or the concept of ecosystem services into a planning process. We need to be adaptive in managing the process and find windows of opportunity to make it happen.

Another aspect is thinking about being “targeted.” A practical approach to avoid being overwhelmed by complexity is targeting and trying to reconstruct cause–effect relationships (Elliott et al. 2020). For example, using tools and models to build knowledge to identify and respond to specific challenges, and then building on that. These are practical strategies and techniques in the planning process to push it to be adaptive.

**L.B.C.:** I got into MSP trying to implement ecosystem-based management, which includes the biophysical, ecological, and human dimensions (McLeod and Leslie 2009). It was impossible to imagine doing it in a way that was not spatial. Spatial management has been developed for marine systems for a long time, for example, with time-area closures in fisheries and decades of efforts in marine-protected areas (Reimer et al. 2020). However, with climate change—and even with events such as El Niño and La Niña or seasonal and interdecadal climate variability—the places where we need to “draw the lines” in MSP will be different.

Most marine spatial plans have been oriented to species and habitats that “sit still” for most of the time, such as coral reefs and kelp forests, or to human uses that are easily mapped into longitude and latitude boxes. For that reason, MSP ends up lacking the flexibility to follow dynamic ocean processes, let alone long-term climate change (Frazão Santos et al. 2020; Rilov et al. 2020). While planners were designing static boxes and planning, ocean users operated the ocean space with real-time technology, often following resources by tracking the shifting positions of oceanographic features (Maxwell et al. 2015). It is then curious that management has taken a more static approach. It may have been because we tried to apply land-use planning (where things sit still) to the sea (where everything moves).

To respond to this mismatch, the idea of dynamic ocean management arose a number of years ago. Dynamic ocean management corresponds to management that changes in space and time at scales relevant to species movements and human use (Maxwell et al. 2015). It combines remotely sensed environmental data with observation data and incorporates static and dynamic oceanographic features into the analysis—while a seamount would stay fixed, an eddy would move around. For example, remotely sensed satellite tags can be placed on marine animals, and corresponding data can be used to map where such animals are likely to be in particular seasons or with climate change (Block et al. 2011).

This approach could allow for the development of MSP at larger scales, such as the open ocean, where species and habitats often move significantly (Maxwell et al. 2020). Designing a static rectangle, fixed into latitude and longitude, is fundamentally wrong for these dynamics. The alternative would be to draw an enormous rectangle to include seasonal and nonseasonal dynamics, which is also not ideal for effective management. For example, in the winter, albatrosses nesting in the Northwest Hawaiian Islands feed about 1000 km away; in the summer, about 2000 km. With climate change, the entire system will be another 1000 km north (Henry et al. 2021).

Another point is that if we can put a planning process into law that allows us to make rapid decisions and be adaptive, that might be something we can sell. We constantly use a dynamic management system for air traffic control to ensure that people travel safely. Based on environmental conditions, decisions about reallocating air space and landing directions are made in near real time. We do not go to a legislator to say, “Let us land from the south”; it is built into the planning process.

**M.L.P.:** Larry is referring to the fact that we can set up the rules for making decisions—rather than prespecifying the decisions—right now. For example, we can establish that some boundaries must follow an oceanographic feature or a particular species. We can set up such a dynamic system as a set of ecological or environmental triggers or guidelines. And we can use dynamic feedback loops and monitoring. That is how we can get, in part, these more dynamic systems into rules and regulations. The critical question is, “What kind of plan, or what set of rules (for adapting) do we need that work well in any of those future scenarios?” In adaptive management, we do not want to reopen the political debate about how to adapt whenever we need to. Instead, we want rules for “if this happens to X, do Y.” That might be based on environmental or social indicators (Ehler 2014). However, we need to decide on those adaptive mechanisms now so they can be implemented quickly when change happens (Murphy 2022). Otherwise, we will be very slow to react.

**J.C.D.:** There is a sense of urgency in dealing with climate change. However, we must proceed cautiously. We do not know what the future holds. Sometimes, what we put in place today is not the right solution in the long term. We can, therefore, make some major wrong decisions today, leading to perverse outcomes. So, we must progress in a way that allows us to address these concerns and make changes. However, decision-makers and politicians do not like it when we keep “chopping and changing” things. That is not what they want when, for example, they are trying to give us the endorsement of a planning process or continued funding. At the same time, we cannot wait to have all the answers to act. So, what we need is an adaptive management approach. To try something, if it works, we build on it; if it does not, we change it and do it better (Allen and Garmestani 2015).

**L.B.C:** The panel is being very thought-provoking and sharing wisdom. In the United States, governments were designed to be deliberative and careful and have checks and balances so that we do not make long-term bad decisions. This works well if the decision-making timeframe matches the time dynamics of the system we are trying to manage. A good analogy or metaphor is, “Imagine you are driving a car on an icy road with a 2-minute delay in both the steering wheel and the brakes; you are going to be in the ditch.” What we see with many complex system dynamics in the real world is that we “end up in the ditch” because the decision-making process is not even close to the time dynamic of the system we are trying to manage. If we were doing MSP and nothing was changing in the ocean (e.g., related to climate change), we could potentially use data from the 1950s, 1980s, or 2000s to set a footprint around which we are planning because things would stay largely in the same place. However, we know that things are changing both on land and at sea (IPCC 2023).

So, in dynamic control system approaches (such as engineering systems), a process is designed to make decisions in near real time, which would support legislative guidance. Only then can decisions be made to match the time dynamic of the system. When we think about it, in the 1900s, humans flew for the first time in powered aircraft. About 120 years later, jets fly themselves. Also, a little over a century ago, cars emerged as devices that humans depend on, and now we have cars that can drive themselves. So, at least in engineering systems, humans have been able to set up adaptive control and management systems that can be automated.

Marine and coastal ecosystems are much more complex than those engineering systems. When I was a kid, if something were relatively easy, people would say, “It is not rocket science”—as if rocket science were difficult. However, rocket science is easy compared to managing marine ecosystems, particularly managing all the people with a “dog in the fight” in marine ecosystems. One key aspect we must push for is that it is excellent if legislators and management bodies want to be deliberative and take their time; however, we will end up in the ditch. Moreover, trying to pull ourselves out of the ditch will be much worse than if we can “speed things up enough to stay on the road.” I know that these metaphors are simplistic. However, we will be in trouble if we do not begin building toward a more rapid decision-making process. We need to choose optimism (Borja et al. 2022) and believe we can do it.

**T.A.:** We also need to harness the ecological and social sciences to the maximum extent possible. To anticipate changes and adapt our management as we move forward. We have the tools and perspectives we need available. We only need to ensure that harnessing the blue economy potential does not occur without using the science we have. We need to be much more aware. We must track climate-related changes and use that data to feed models to anticipate future changes. We need to build our MSP processes and plans so that management can generate the necessary information. This way, we can know where we are going, the pressures on ecosystem functioning, and how much we can use the ocean without compromising long-term sustainability.

**J.C.D.:** Following up on Tundi's point, we must assess and evaluate. Only by monitoring effectively can we know what is working and what is not (Carneiro 2013; Ehler 2014; Stelzenmüller et al. 2021). However, monitoring also takes resources. More than just monitoring, there is a need for publicly reporting the monitoring results so that the public understands why managers are doing what they are doing. All this adds to the complexity of the issue.

## Ocean health as the foundation for sustainable MSP

Here, we dive into aspects that have prevented ocean health from being truly considered as the foundation for sustainable MSP. We further explore solutions to overcome identified challenges, particularly the need to engage with decision-makers, resource users, and the public. The need for ocean optimism is highlighted.

### Do we need to rethink the role of marine conservation and ocean health in MSP, particularly under the challenges of a changing climate?

*Yes. Under a changing climate, we must move beyond the old and false dichotomy between conservation and development and shift the conversation towards developing MSP that ensures ocean health for people's well-being. An equity lens is identified as a new entry point for such discussion*.

**T.A.:** Climate change is already affecting ecosystem health, species distribution, and the levels of use we can enjoy from ocean resources and space. These factors are already adding to the overall stressors affecting the marine environment and how humans can benefit from the marine environment. We need to think about what that means in terms of requiring humans to hold back consumption and to determine the areas where we will say no to ocean development. With the present rush to develop an ocean economy (Golden et al. 2017; Bennett et al. 2019), planning initiatives have focused more on reducing conflicts and maximizing blue growth. However, we must be more careful to ensure that conservation is the foundation for everything we do, all the space allocation decisions we make, and how we determine what use levels are appropriate and where. We can all agree that the baseline strategy moving forward must focus on conserving ecosystem functioning and health, thus allowing us to continue to reap the benefits. Most people would agree that that is our goal—even if we describe it differently when addressing different audiences. However, we have much work to do in terms of changing the perceptions of the public and decision-makers.

When we talk about conservation and MSP, it is not about setting out a marine spatial plan for protected areas or, more specifically, no-take areas (Grorud-Colvert et al. 2021). It is about creating plans allowing ocean use while maintaining ecosystem health. However, that will also involve defining areas to be strictly protected, targeting vulnerable species, or particularly important ecosystem processes taking place—all of which will be further impacted by climate change. So, we need to take what we need from conservation to promote equitable and sustainable ocean use while not locking in a debate of “us” versus “them” or “nature” versus “humans.” We must not allow that old and false dichotomy to rise back to the surface (Kyriazi et al. 2013; Reimer et al. 2023). It is an unconstructive partisanship to make divisions between resource users and conservationists or between people and nature.

We see many conservationists providing good information, good models, and a good rationale for climate-smart and sustainable marine spatial plans. In many places worldwide, we are building ways to generate and integrate information and knowledge into plans and make people more confident about possible futures (Haasnoot et al. 2013). However, there is also the situation where conservationists are using systematic conservation planning tools and artificial intelligence to support informed planning, and where such a process is almost entirely separate from MSP, *vis-a-vis* the ocean economy and blue growth. There is a “marginalized” conservation community working very hard to create a robust approach for protecting “the golden goose” while allowing sustainable ocean use and also focusing on equity issues (Bennett et al. 2021a; Bennett et al. 2021b).

So, we need to quickly shift the conversation around what MSP can be and do. We must also work on communication in the conservation community because the future cannot be a battle between “us” and “them.”

**E.G.:** I agree with Tundi; it is a matter of shifting how we build the conversation with decision-makers. Supporting ocean health through MSP is particularly critical in the age of climate change. We will only move forward if we stop confronting conservation with development and stop using the same narrative about hard versus soft sustainability (Qiu and Jones 2013). The discourse we now find more frequently and need to adopt is that we care for nature because we depend on it.

We are trying to build narratives together, and there are many good examples. In Europe, we have a new communication about the ecological transition, in which nature conservation is not separated from economic benefits (European Commission 2019). Also, in the South Pacific, the Marae Moana (“Sacred Ocean”) initiative for the holistic ocean management of the Cook Islands incorporated indigenous principles in managing the surrounding ocean space (Durbin 2018). The latter was based on the premise that “we depend on the marine resources.” This premise allowed local leaders, local communities, and key actors of the plan to drive the importance of the sea for the communities first, when building the visions for the plan. This vision was central to leading the plan towards a positive narrative of humans in a healthy and productive ocean. Following this narrative, decision-makers, communities, and scientists have worked together in an effective way (Pennino et al. 2021).

**L.B.C.:** I agree we need to include conservation components of MSP. The fundamental problem is that marine conservation started in this venue with protected areas, which were all about telling other people they could not use part of the ocean. Saying, “We should just get rid of all global fisheries in the open sea,” is not a very effective “conversation starter” for many people. We must look for opportunities to work with people responsible for development in coastal areas and exclusive economic zones and try to work conservation into their plans.

Most ocean uses depend upon a healthy ocean and the proximity of healthy marine ecosystems to land. We often think about fisheries and how they require a healthy ocean (Sumaila et al. 2021). However, tourism, the number one economic driver in most coastal areas worldwide (Arabadzhyan et al. 2021; Smith et al. 2023), also requires a healthy ocean (Friedman et al. 2022; Evans et al. 2023). Indeed, tourists do not want to go and see a dead ocean; they want to see a healthy one (Agardy 2018; Friedman et al. 2022). Even in marine transportation, we may take a vessel or move freight on a ship over a dead ocean, but we still need to worry about whether or not we will run into a blue whale.

We should remind those in business that they need a healthy, functioning ocean to thrive. We must put it into those utilitarian terms that make sense to them—painful it may be—and showcase how such businesses have an implicit interest in ocean conservation. Instead of framing conservation as “another sector jumping for the cookie jar.” Biologists often feel ill-equipped and not adequately trained to have these conversations. However, having such conversations and co-creating problem definitions and pathways to solutions is what we need to do. Not because it is easy (we can imagine it being easy, yet it is actually quite challenging). But because we must.

**M.L.P.:** There has been an interesting switch in how we think about ecosystem services and contributions to people from nature (Díaz et al. 2018). We are moving away from thinking about these from an income perspective, “Let us value the flow of benefits out of the ocean,” to a wealth perspective, “Let us value the state of ocean ecosystems for what they can continue to produce through time.” For example, a wealth perspective values the fish in the ocean because they represent the potential for future fisheries or tourism. An income perspective would only value the flow of fish that we catch. The wealth perspective is now what the World Bank is trying to use (World Bank 2021b). Economists like Partha Dasgupta and Eli Fenichel have helped push such a perspective forward (Fenichel and Abbott 2014; Dasgupta 2021). This perspective also provides an additional justification for conservation, which can be helpful. It is closer to a more realistic valuation.

**E.G.:** Following up on previous comments, one way to move forward is to think about how MSP can contribute to ecosystem health and human well-being through the equity lens. Equity should be the “entry point” for MSP by connecting the benefits provided by ecosystems in healthy conditions with communities and economic actors benefitting from those ecosystems—and with such communities and economic actors potentially affecting the same ecosystems. Unraveling these connections is essential to building an equitable and healthy ocean while distributing benefits and controlling impacts. With this approach to MSP through the equity lens, we can reconcile ecosystem and human health, supporting a blue economy based on a just and productive ocean. Through equity, we can reconcile conservation and blue economy. Moreover, this approach embraces ocean optimism toward positive solutions (Borja et al. 2022).

### Must we then build a common narrative to engage more deeply with decision-makers, resource users, the public, and other stakeholders?

*Yes. The need for effective communication among sectors, as well as the importance of having all voices represented in decision-making and of managing expectations, is highlighted here*.

**T.A.:** We need to convince the public and decision-makers that conservation is not a “special interest”; it is the strategy that allows for the best, most equitable use, and the broadest benefit flow coming from the ocean in the future. We must look for opportunities to highlight approaches working well, showing how an investment over time reaps benefits. However, a lot of MSP initiatives around the world are very frontloaded. That is, there is a significant investment of time, energy, and money into developing a plan as if such plan were the endpoint or goal. At the same time, there is very little investment in monitoring, evaluation, and making changes toward adaptive ocean management (Carneiro 2013; Stelzenmüller et al. 2021). We must shift the conversation and have decision-makers understand why conservation-oriented MSP benefits everyone. Such an approach will lead us to a truly sustainable blue economy—instead of “blue growth” masquerading as a sustainable blue economy (Bennett et al. 2021a).

**J.C.D.:** Indeed, while conservation is a critical use of the ocean, we must balance all other uses. Moreover, this must be done “with” the public rather than imposed upon them—if they are ever to comply. There is the problem of top-down or frontloaded approaches, as Tundi mentioned before. We have seen this in previous planning exercises (Merrie and Olsson 2014; Flannery et al. 2018). If MSP is imposed upon a community—particularly fishermen—and people do not understand why or how it works, they will not comply with the process, and we will have wasted our time. We need to bring the public and decision-makers on the journey. It is a difficult journey because we do not know exactly where it will lead and where we will get to. However, it is something we cannot “shy away” from. It was mentioned that we need to educate legislators to agree to a process; this is precisely where we need to go. However, the latter will be a challenging task—easier in some parts of the world but not easy globally.

**T.A.:** The reality check Jon brings to the panel is valuable because it is based on extensive experience dealing with decision-makers, managers, and people's perceptions. Indeed, we must not raise unrealistic expectations. At the same time, we need to be very convincing about investing in these processes that will help us overcome some of the knowledge gaps. Processes that are every bit as much about developing the “optimal plan” for the blue economy and blue growth as they are about setting up a process in which we are careful, aware, and deliberate in the way we use the ocean space and its resources.

**L.B.C.:** I believe part of the conflict goes way back to marine conservationists looking at people doing coastal development as “the enemy.” When MSP emerged in the United States 20 years ago (Ehler 2021), a marine conservation colleague said, “you know what I want to get out of MSP? More totally protected marine areas.” The development community saw through it immediately, saying that MSP was “marine-protected areas spelled differently.” We are trying to start a cooperative, co-creation approach from a background of antagonism and with a high level of distress—which we must overcome. The integrative planning processes that have taken place ever since have been much more engaging, to the point where some conservationists feel they are losing their seat at the table—as this becomes not “marine” but “maritime” spatial planning (Rilov et al. 2020; Kirkfeldt and Frazão Santos 2021). So, most of the work we have to do is actually about interactions with decision-makers and stakeholders.

The benefit of coming to the table in an MSP process is that everyone can participate and strongly represent their interests (Gopnik et al. 2012). Many people are concerned about having little space for everybody, but the ocean is vast. For example, Palau has more ocean space than land space (Gruby et al. 2017). So, while we should not yield conservation interests to development interests, we must have a conversation, not just an antagonistic relationship.

Suppose we do all the science we need (i.e., our techniques properly incorporate climate change, human dimensions, and equity issues). We do it all. Would it then be easy to negotiate a plan or a process with legislators, stakeholders, and the various interests, including conservationists? No, it never becomes easy. It is still going to be hard. That is why we need environmental negotiators and lobbyists to help with such conversations. It is where we are failing. If we want to reach sustainable development, improving such conversations is a critical task—one we have not done very well up to this point.

**T.A.:** Adding to what Jon and Larry said, however, many times after saying we want to promote sustainable, appropriate, and equitable ocean use—having “checked the box”—people then turn to promote use that is not appropriate, further undermining ecosystems and their resilience. Many times, there is an excessive emphasis on blue growth. Indeed, most emerging plans focus on compatible uses, reducing user conflicts, and unlocking the blue growth potential (Merrie and Olsson 2014). In extreme cases, the latter may lead to ocean grabbing, where powerful industries gain access to places at the expense of coastal communities (Bennett et al. 2021a).

For all these reasons, as Larry mentioned before, co-creating plans in a way that ensures all existing values are considered is fundamental—especially for coastal communities that are highly dependent on the ocean. That is what good marine spatial plans allow us to do. They allow us to keep doors and future options open. We must take the climate science we have and apply it to anticipate possible futures and plan for the best ways to use the ocean in such imaginable futures (Merrie et al. 2018; Nash et al. 2022). However, never letting go of the conservation element. Because as soon as we say, “This is not about protected areas,” either we insist that MSP must also include some strict protections—for some species and habitats—or we will lose everything. We will hand it over to the big industrial users. That would be a disaster for coastal communities, ecosystem health, and, to be honest, our future as a species.

**A.H.C.:** To follow up on what others have said, I think there has always been this mistrust, the idea of a historically antagonistic relationship between the conservation and development sides. However, much effort is being put into bridging the two sides, with many people working on it. In addition, more people are joining the conversation and starting to recognize that conservation will only work if we bring the two sides together. A key point here is that we need incentives for the different sides to come together and effectively communicate.

For example, the Food and Agriculture Organization has been working to identify how spatial management targets of the Convention on Biological Diversity can be brought in and how fisheries management efforts can be incentivized to use effective area-based fisheries management strategies to contribute to biodiversity conservation (FAO 2022). Extractive resources, such as fisheries, are dependent on healthy ecosystems, as biodiversity and ecosystems are needed to make fisheries successful. By bringing fisheries, conservation, and many other sectors’ interests—such as defense or shipping—to the table, we are incentivizing them to get beyond some of that historical antagonism. Incentivizing them to figure out, “How can we do it together?”

Unfortunately, there is still much pushback. We face it every week when discussing the Convention on Biological Diversity and how the fisheries sector fits into it. Many people on the conservation side often say, “No, we just need to close everything up,” not considering community livelihoods or food security issues (Bennett and Dearden 2014). Slowly but surely, we will get there. We need to ensure that enough people continue focusing on working together, that opinions are shared, and that different viewpoints and perspectives are considered.

**J.M.R.:** Others mentioned earlier that we need a new narrative on how to talk about conservation and MSP and move beyond conservation as a “special interest”—which is really important. However, who would be the audience for such a new narrative? To whom are we talking? And will that change how we communicate to be more effective?

This is important to consider. In a recent qualitative analysis of MSP case studies where we investigated how, broadly, conservation was captured by MSP in practice, we did not see finer principles of conservation permeating MSP (e.g., principles like maintaining ecological connectivity, ecosystem function, and being long-term) (Reimer et al. 2023). So, in the same way that we make MSP climate-ready or climate-smart, can we make MSP “conservation ready” or conservation-smart?

**T.A.:** I believe it is not “one” audience but concentric circles of audiences. It starts with decision-makers at the level of government agencies, developing MSP and making decisions on space allocation, but it must go much beyond that. Decision-makers have their constituencies. Even if they are not political appointees, they still represent a particular worldview. We can have a wide variety of voices, some advocating for “not eating fish,” “closing down the whole ocean to fishing,” or “stopping tourism development.” However, those whom we really need to engage are the resource users. We must make the case to all ocean stakeholders that investing in and protecting a healthy, functioning ocean benefits “them.” And that MSP allows us to do that effectively—particularly integrative, cross-biome, forward-looking MSP. As much as we may not like to commodify nature, this resonates with people because of human nature and how humans think about things. The conversation then reaches an even wider circle: the general public. So, first and foremost, environmental agencies; second, the people invested in ocean use (i.e., the business community); and third, the wider public.

**E.G.:** In addition to what Tundi said, an interesting audience to consider is the private sector and investors in blue innovation (i.e., those who think more broadly and see opportunities). More than strictly thinking only about decision-makers. For example, we mentioned blue carbon sequestration and blue carbon initiatives (e.g., Tavonvunchai 2022). While these initiatives are conceived to support conservation, they use a broader, different perspective. In effect, many initiatives have been created by doing business on innovative ideas supporting conservation. However, many uncertainties still persist in how to set and implement these initiatives (e.g., with respect to blue carbon) (Macreadie et al. 2022). So, the narrative opens especially to the private market.

**M.L.P.:** From a planning perspective, when we think about many objectives simultaneously, we end up with something much closer to a win-win solution. That has been shown many times (e.g., White et al. 2012). However, to get there, we must ensure that all voices are represented at the table. However, MSP is often a fairly technocratic process (Ehler and Douvere 2009; Flannery et al. 2018). There may be public meetings, but people need to be able to attend them and speak up. Also, sometimes there may be too many meetings, or essential constituents may not be able to participate. The environment itself cannot participate, which is why having advocates for ecosystems is so important (Calado et al. 2012; Gopnik et al. 2012). The same goes for marginalized social communities (Zuercher et al. 2022). People may not have the time to go from work to meetings and speak up. An essential part of the process is thus thinking about how to represent the critical actors that cannot participate directly.

**J.C.D.:** Following up on Malin's comment, a false expectation we must not promote is that MSP will always result in win-win situations (Jones et al. 2016; Flannery et al. 2018). In large-scale MSP or planning of any sort, the concept of a win-win is almost impossible. We should be saying that everybody will have to give up something, so it is more of a “lose-lose,” but still, people need to be involved in decision-making as much as possible. Another issue pertains to integration. We cannot talk about coastal waters in the same way as we do about national offshore waters, let alone the high seas or going across different nations. So, developing a legislative framework covering multiple jurisdictions is undoubtedly complex (Scholten 2019); however, there are precedents (e.g., the complementary zoning approach applied in both federal and state waters in the Great Barrier Reef) (Day et al. 2019). These are enormous challenges. This is not to say that nothing will ever work, only that we must be realistic about some of the complexities facing dynamic MSP.

**M.L.P.:** Jon made an excellent point about win-win. Indeed, no one wins to the same extent they would win individually. However, we end up with a solution where everyone has more than they would if they were not at the table. We get closer to that efficient solution that works well for everyone.

**J.C.D.:** Going back to the point on false expectations, we must be clear that none of the things we discussed here today will fix climate change. To do so, we must reduce global emissions (IPCC 2023). Otherwise, all we discussed is excellent, but it will make no difference. We must keep reminding the public and decision-makers that we have to work on many fronts, and the emissions one is major. However, many people are pushing against it (Flannery 2019), at least in my part of the world [Australia]. As planners and scientists, there is the risk of looking at things through a single lens when the reality of the problem is much broader and more complex. We must work on these things as we certainly cannot shy away from them. Many challenges are ahead of us, but we must try to make it work.

**L.B.C.:** Someone pointed out before the “complexities” the human mind can handle using a simple metaphor: when we are taught our phone number, we are given three digits and then four digits; for our social security number, three digits, two digits, four digits; this is because humans cannot remember seven digits without somehow breaking them down into clusters. We must remember that we have fundamental limitations whenever patting ourselves on the back for our brilliance. So, if we are to get to the point where we equitably and rationally use exclusive economic zones, or even beyond, the open ocean, we need to start thinking about that now.

What skill sets do we need to put together to begin addressing that? Because the alternative of saying, “It is too hard, we cannot do it,” does not work. In such a case, we will end up with autocracy and absolute power going forward. We will not have conservation considered; we will not have the needs of local people or coastal resource-dependent communities considered. So, it is fair to say, “Yes, it is difficult, but we need to think about it now.” We need to think about how we will take the first step, even if we can only hope to get there in years to decades. Optimism keeps us engaged (Borja et al. 2022). If we sink into pessimism, we will go to hopelessness next. And we cannot afford to be hopeless right now with marine conservation and management.

## Post hoc considerations

Having debated critical topics for sustainable ocean planning and conservation in the age of climate change (i.e., main challenges posed by climate change to MSP, integration of climate-related knowledge, the need for dynamic and adaptive planning, the importance of ocean health as the foundation for MSP, and building common narratives to engage stakeholders and decision-makers), we recognize that not all relevant voices and perspectives are represented in this discussion. As the session was organized as part of the People & the Sea scientific conference, it primarily reflects perspectives from scientists and practitioners (Supplementary Table 1). Perspectives from the broader landscape of stakeholders that are to be involved in ocean planning and management (e.g., local communities, NGOs, and industry) are missing, which naturally influences the ideas that emerged in the discussion.

An additional key point mentioned in the session, but not thoroughly discussed, was the fundamental importance of accounting for social change, equity, and power imbalances (Bennett et al. 2021b; Crosman et al. 2022) when developing MSP initiatives. MSP needs to prioritize the needs of local communities in terms of livelihoods, food security, and cultural values, and engage them in the co-development of solutions for ocean use and conservation. Only by doing so can MSP effectively deliver sustainability outcomes in the long term. Indeed, reinforcing the importance of social knowledge, equity, and change in co-developing marine spatial plans has been identified as one of the key components of climate-smart MSP (Frazão Santos et al. 2024b).

Similarly, the aspect of climate mitigation was only mentioned briefly in the discussions. Identifying ocean-based climate solutions and prioritizing space allocation to support climate mitigation and adaptation actions is another key component of climate-smart MSP—and one that has been advocated for several years (Frazão Santos et al. 2020, 2024b). Climate-smart marine spatial plans can, for example, significantly support the potential for renewable energy production in the ocean or identify blue carbon ecosystems and prioritize the allocation of space for their restoration and conservation accordingly. MSP can also support climate adaptation actions, by supporting both social and ecological resilience to climate-related impacts (Frazão Santos et al. 2024b).

The development of climate-smart MSP is “on the move,” with increasing awareness being raised and benefits being recognized both for national jurisdictions around the globe (UNESCO and European Commission 2022) and far beyond (i.e., high seas; Frazão Santos et al. 2024a). Planning for climate change has never been as relevant and crucial as it is today. With the upcoming 16th meeting of the Conference of the Parties to the Convention on Biological Diversity (COP 16) taking place later in October 2024, and expectations to have the United Nations Agreement on the conservation and sustainable use of marine biological diversity of areas beyond national jurisdiction ratified by enough countries so that it goes into force in 2025, insightful discussions around the development of sustainable and climate-smart ocean planning and governance approaches are needed (Frazão Santos et al. 2024a; Hannah et al. 2024) and must be brought under spotlight.

## Conclusions

Without healthy and productive marine ecosystems, humans will be unable to ensure long-term socioeconomic benefits and well-being from the ocean (Díaz et al. 2018; Allison et al. 2020). An integrated approach, based on a healthy ocean and with adaptive management driving constant amendment and improvement, is vital to face climate-induced changes, increase social–ecological resilience, and reduce climate-related impacts and other local human stressors (Frazão Santos et al. 2020, 2024a).

Within this article, we have asked important questions about the challenges and opportunities that emerge from developing MSP initiatives that foster full-spectrum sustainability under a changing ocean. We hope that by sharing a deep dive into these discussions we further contribute to a much-needed paradigm shift of how we perceive the sustainable use and conservation of our ocean. Emerging from these discussions, a number of key points should be kept in mind as we navigate the expansion of MSP around the globe under environmental and social change:

- A changing ocean will require dynamic boundaries and adaptive solutions to complex problems;
- Ocean health must be prioritized as the foundation for MSP;
- We must develop future looking, proactive plans that account for where ocean resources and users will likely be in decades or centuries to come;
- Monitoring and evaluation are fundamental to knowing what is working and what is not in our plans;
- While not easy, it is fundamental to ensure legal flexibility and legal certainty in MSP;
- A common narrative built with decision-makers, stakeholders, and the public will overcome the existing false dichotomy between nature conservation and human development;
- MSP needs to prioritize the needs of local communities in terms of livelihoods, food security, and cultural values;
- Care should be taken not to raise false expectations about what MSP can deliver;
- Engaging in ocean optimism will lead to more engagement than emphasizing the negative; and
- MSP can help solve ocean issues, but only if we **act *now***, before it is too late.

## Supplementary Material

obae037_Supplemental_File
