# Supplementary material for: Ocean Planning and Conservation in the Age of Climate Change: A Roundtable Discussion
Source: Integr Org Biol. 2024 Sep 26;6(1):obae037. doi: 10.1093/iob/obae037 (PMC11495413; doi:10.1093/iob/obae037)
Supplement: obae037_Supplemental_File [file obae037_supplemental_file.docx]

Supplementary data for

Ocean planning and conservation in the age of climate change: a roundtable discussion

**Supplementary Table 1**

**Supplementary Table 1.** Background of all people actively involved in session 3.161, “Marine Spatial Planning and Ocean Conservation in the Age of Climate Change”, at the 11th MARE People & the Sea Conference (chairperson, invited speakers, and active participants).

| **Name** | **Background and experience** |
| --- | --- |
| Catarina Frazão Santos  (chairperson) | Dr. Catarina Frazão Santos is an Invited Assistant Professor at the University of Lisbon (Department of Animal Biology, Marine and Environmental Sciences Center) and Honorary Research Associate at the University of Oxford (School of Geography and the Environment). Her research focuses primarily on the challenges of developing sustainable ocean planning and governance under global environmental and social change. Her publications have appeared in *Science* and several *Nature* journals, among others, and have contributed to bridge the science-policy gap. Catarina is founding Editor-in-Chief of the *Nature npj Ocean Sustainability* journal and awardee of the European Research Council (ERC Starting Grant, project PLAnT “Planning for sustainable ocean use in Antarctica under global change). She has a Ph.D. in Marine Sciences (co-directed by Duke University), a M.Sc. in Environmental Management, and a degree in Marine Biology from the University of Lisbon. |
| Tundi Agardy  (invited speaker) | Dr. Tundi Agardy works at the interface between science and policy in marine systems around the world. She founded Sound Seas to do this independent work, and also holds positions with Forest Trends, as MARES Director (a program looking to protect Marine Ecosystem Services through Payments for Ecosystem Services markets), and at Baird & Associates, where she is Caribbean Environment Lead. Tundi is an internationally renowned expert in marine conservation, with extensive field and policy experience in Africa, Asia, the Caribbean, the Mediterranean, North America, and the Pacific. Her major research interests and publications focus on coastal and marine planning, ocean zoning, marine protected areas, ecosystem services, and marine ecosystem-based management. She received her Ph.D. in Biological Sciences and Master’s in Marine Affairs from the University of Rhode Island. |
| Larry B Crowder  (invited speaker) | Larry Crowder is the Edward F. Ricketts Provostial Professor of Marine Ecology and Conservation in the Oceans Department at the Stanford Doerr School of Sustainability, senior fellow at the Woods Institute for the Environment, and a professor, by courtesy, of biology at Stanford University. His research focuses on predation and food web interactions, population and food web modeling, small-scale fisheries, dynamic ocean management and interdisciplinary approaches to marine conservation and policy. He was Principal Investigator for large interdisciplinary research projects including the OBIS SEAMAP (Spatial Ecological Analysis of Megavertebrate Animal Populations), and Project GLOBAL (Global Bycatch Assessment of Long-Lived Species). He has also directed and participated in a number of research, analysis, and synthesis groups at the National Center for Ecological Analysis and Synthesis (NCEAS) and for the National Research Council's Ocean Studies Board. His recent research has focused on marine conservation, including research on bycatch, spatial ecological analysis, sustainable seafood, ecosystem-based management, marine spatial planning, and governance. |
| Jon C Day  (invited speaker) | Dr. Jon Day commenced his professional career in Australian national parks (1975-86). In 1986, he joined the Great Barrier Reef Marine Park Authority (GBRMPA), the federal agency responsible for the Great Barrier Reef. For the following 28 years, Jon was involved in many aspects of planning and managing the Great Barrier Reef, including seven years in field management and 16 years as one of the Directors at GBRMPA. As a Director, Jon was variously responsible for biodiversity conservation, park planning, heritage (particularly World Heritage), Indigenous Partnerships, the GBR rezoning (for which he was awarded a PSM) and commencing the first 5-yearly Outlook Report. Jon retired in 2014 to undertake a post-career PhD at the ARC Centre for Coral Reef Studies at James Cook University. The Climate Vulnerability Index (CVI), co-developed by Jon, is being increasingly applied globally, particularly in World Heritage properties. |
| Malin L Pinsky  (invited speaker) | Malin Pinsky, Associate Professor at the University of California Santa Cruz, is a biologist with expertise in the adaptation of ocean life to climate change, including ocean conservation. His more than 100 publications have appeared in *Science*, *Nature*, and other journals, and his research has been covered by the *New York Times*, *Wall Street Journal*, and *BBC*, among others. He is a Fellow of the American Association for the Advancement of Science, an Earth Leadership Fellow, and an Early Career Fellow of the Ecological Society of America. He was named one of Science News’ ten scientists to watch in 2019. Malin serves on advisory boards for the Beijer Institute of the Royal Swedish Academy of Sciences, the non-profit Oceana, and the Chewonki Foundation. He has a Ph.D. in Biology from Stanford University and an A.B. in Biology and Environmental Studies from Williams College. He grew up exploring tidepools and mountains in Maine. |
| Elena Gissi  (invited speaker) | Dr. Elena Gissi is a Senior Researcher at the National Research Council, Italy, and Adjunct Professor at the Polytechnic University of Marche, Italy. Her research focuses on modelling the responses to climate change of marine life at multiple levels of biological organization, to incorporate this knowledge in marine spatial planning (MSP) for adaptation and mitigation. She has 20 years of research experience in producing and integrating ecological insights in decision-making processes, with an expertise spanning MSP, conservation planning, and community ecology. She was a Marie Skłodowska Curie Fellowship with the project MEDIX “Marine Environmental DynamIcs and seX-based analysis for climate change adaptation in marine spatial planning”, for which she was a Visiting Scholar at Stanford University (2020-2023). At Stanford, she developed the foundation for sex analysis and the gender dimension of research in marine biology and environmental sciences. Elena designed and contributed to the first tests of implementing MSP in the Mediterranean, and she is appointed as an expert in the planning team of the Italian MSP process. |
| Amber Himes-Cornell  (participant) | Dr. Amber Himes-Cornell is a Fishery Officer of the Fisheries and Aquaculture Division of the UN’s Food and Agriculture Organization (FAO) and is based in Rome Italy. At FAO, her work is focused on area-based management of fisheries and aquaculture, spatial planning, coastal community vulnerability and resilience, social transformation, socio-economic impacts of fisheries and aquaculture management, climate change adaptation strategies, the socio-economic aspects of marine ecosystem services, and valuation of ecosystem services. |
| Julie M Reimer  (participant) | Dr. Julie Reimer is a Senior Policy/Economic Advisor at Fisheries and Oceans Canada, National Marine Spatial Planning Program, and Invited Assistant Professor at the Dalhousie University (Faculty of Science - Marine Affairs Program). She is an interdisciplinary researcher with experience in academia, government, and non-governmental organizations. Through her work, she fosters collaborative partnerships to develop conservation and sustainability solutions for the ocean and ocean users. She has a Ph.D. in Geography from the Memorial University of Newfoundland (St. John's, NL) and a Master of Marine Management from Dalhousie University. ​ |
